# Identification of IFN-Induced Transmembrane Protein 1 With Prognostic Value in Pancreatic Cancer Using Network Module-Based Analysis

**DOI:** 10.3389/fonc.2021.626883

**Published:** 2021-03-22

**Authors:** Lingyun Wu, Xinli Zhu, Danfang Yan, Mengmeng Tang, Chiyuan Ma, Senxiang Yan

**Affiliations:** ^1^Department of Radiation Oncology, The First Affiliated Hospital, Zhejiang University School of Medicine, Hangzhou, China; ^2^Department of Pathology, The First Affiliated Hospital, Zhejiang University School of Medicine, Hangzhou, China; ^3^Department of Orthopedic Surgery, The Second Affiliated Hospital, Zhejiang University School of Medicine, Hangzhou, China

**Keywords:** IFITM1, IFN-induced transmembrane protein 1, pancreatic cancer, functional interaction network, prognostic biomarker, tissue microarray detection

## Abstract

Despite improvements reported in diagnosis and treatments in recent decades, pancreatic cancer is still characterized by poor prognosis and low survival rate among solid tumors. Intensive interests have grown in exploring novel predictive biomarkers, aiming to enhance the efficiency in early detection and treatment prognosis. In this study, we identified the differentially expressed genes (DEGs) in pancreatic cancer by analyzing five gene expression profiles and established the functional modules according to the functional interaction (FI) network between the DEGs. A significant upregulation of the selected DEG, interferon (IFN)-induced transmembrane protein 1 (IFITM1), was evaluated in several bioinformatics online tools and verified with immunohistochemistry staining from samples of 90 patients with pancreatic cancer. Prognostic data showed that high expression of IFITM1 associated with poor survival, and multivariate Cox regression analysis showed IFITM1 was one of the independent prognostic factors for overall survival. Meanwhile, significant correlations of the expression of IFITM1 and the infiltration of immune cells were found by TIMER. Furthermore, a higher level of IFITM1 was assessed in pancreatic cancer cell lines compared to normal human pancreatic duct epithelial cells, and silencing IFITM1 in tumor cells remarkedly inhibited cancer tumorigenicity. Collectively, our findings suggested that IFITM1 might have promising utility for pancreatic cancer.

## Introduction

Pancreatic cancer is expected to be the second leading cause of cancer-related deaths in developed countries by 2030, and is characterized as highly invasive and metastatic as well as extremely resistant to chemo-radio-therapy (1). Despite some efforts made on adjuvant therapeutics recent years, the clinical mortality ratio remains elevated to date (2). The 5-year survival rate was reported <10% with an estimated 432,242 deaths occurring worldwide in 2018 (3). Because of this poor prognosis, better identification of clinical biomarkers useful for making therapeutic decisions and developing targets for innovative drugs are urgently needed in this complex and heterogeneous mutational landscape.

The human interferon (IFN)-induced transmembrane protein 1 (IFITM1), also named Leu13 or CD225, is a 17-kDa cell-surface membrane protein in the IFN-stimulated genes (ISGs) protein family along with IFITM2 and IFITM3. Since it was first discovered in neuroblastoma cells in 1984, it has been identified participating in various biological processes (BPs) including cell proliferation and adhesion and being important in immunity and antiviral activities (4, 5). Particularly, more and more studies have confirmed that IFITM1 is overexpressed in numerous human cancers during the last decade, such as lung, gastric, colorectal, and ovarian cancers (6–9). Furthermore, the positive IFITM1 expression was illustrated in correlations with poorer prognosis in diverse tumors (10, 11). Hence, it is very likely that IFITM1 may also hold hitherto undiscovered value in pancreatic cancer and might be further associated with prognosis of patients with pancreatic cancer.

In this study, we investigated gene expression profiles from Gene Expression Omnibus (GEO) repository to screen out common DEGs between pancreatic cancer and normal pancreatic tissues and analyzed them by establishing a protein functional interaction (FI) network. Functional network-based modules were constructed, and biological pathways were analyzed afterward. It was confirmed that IFITM1 played a vital role in pancreatic cancer by analyzing the clinical and pathological characteristics of 90 patients with pancreatic cancer and their survival rate as well as by verifying through several bioinformatics online tools. Our study suggested that IFITM1 may have promising clinical utility for prognostic stratification, and combining IFITM1 expression profiles systematically with clinical characteristics of patients is promising to be effective for developing treatment.

## Materials and Methods

### Research Tools

**Figure 1:**
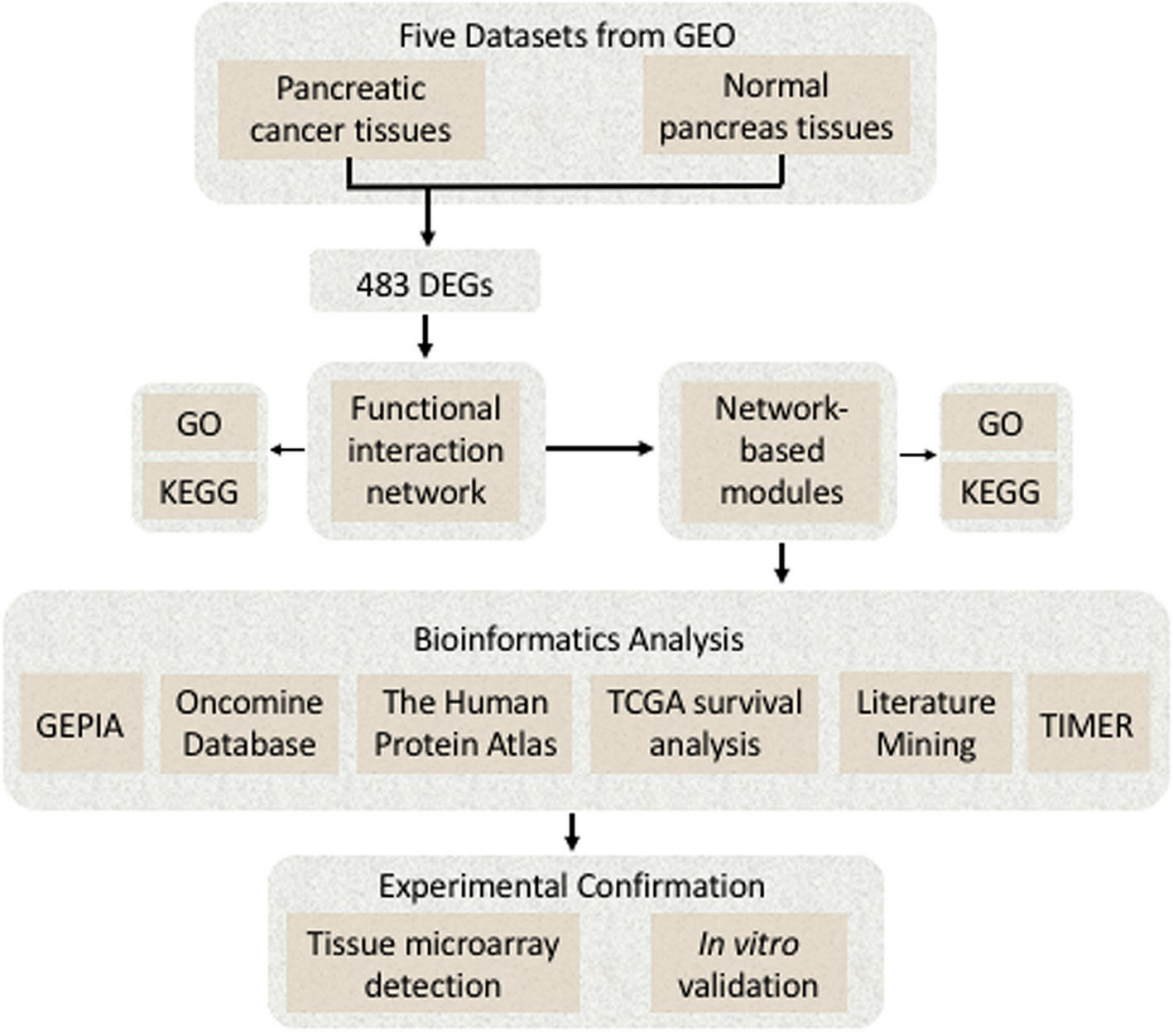
A detailed flowchart of identifying and verifying the targeted differentially expressed genes (DEGs) associated with pancreatic cancer.

(1) BRB-Array Tools (version 4.6.0; http://linus.nci.nih.gov/BRB-ArrayTools.html) were used to analyze the DEGs (12); (2) the Cytoscape software (version 3.7.1; http://www.cytoscape.org) was applied to construct FI network and the network-based functional modules; (3) the FunRich (version 3.1.3; http://www.funrich.org) was used to construct biological pathways (13); (4) the Omicshare online database (https://www.Omicshare.com/), a commercial database based on the Database for Annotation, Visualization, and Integrated Discovery (DAVID), was used for the visual analysis of Gene Ontology (GO), and Kyoto Encyclopedia of Genes and Genomes (KEGG) was used for enrichment analysis (14); (5) the MultiExperiment Viewer (MeV) application (version 4.9.0; http://mev.tm4.org/) was used to generate the heat map; and (6) some bioinformatics online tools such as the Oncomine database (https://www.oncomine.org/), the Human Protein Atlas (https://www.proteinatlas.org/), the interactive web application Gene Expression Profiling Interactive Analysis (GEPIA) (https://www.gepia.cancer-pku.cn), and the TIMER database (https://cistrome.shinyapps.io/timer/) were used. A detailed flowchart of identifying the targeted genes associated with pancreatic cancer is shown in Figure 1.

### Patient Samples Preparation

Between 2004 and 2008, a total of 90 human pancreatic cancer specimens were collected from the Taizhou People's Hospital of China for tissue microarray (TMA) detection, 60 of which were paired nonmalignant tissue. All the specimens were stored at the Biobank Center of National Engineering Center for Biochips at Alenabio and Shanghai Outdo Biotech Company, China. All the patients received no preoperative anticancer treatment or postoperative adjuvant chemo-radio-therapy. Histological differentiation grade and disease stages were classified according to the American Joint Committee on Cancer (AJCC) TNM Classification (7th Edition). The follow-up time was recorded from the date of the surgery to the date of death or last visit to the clinic. The use of pancreatic cancer samples and clinical data were reviewed and approved by the Ethical Committees of the National Engineering Center for Biochips at Shanghai along with the Taizhou People's Hospital. Informed consent was written in accordance with the Declaration of Helsinki from all participants.

### Microarray Data

To investigate biological characteristics of genes differently expressed between pancreatic cancer and normal pancreatic tissues, five data sets were retrieved from National Center for Biotechnology Information (NCBI)-GEO (http://www.ncbi.nlm.nih.gov/geo/) for the analysis, including GSE101448 (18 with pancreatic tumor and 13 non-tumor pancreatic tissue samples), GSE62165 (118 pancreatic tumor samples and 13 control samples), GSE62452 (69 pancreatic tumor and adjacent non-tumor tissues), GSE41372 (matched pancreatic ductal adenocarcinoma samples from 15 patients), and GSE28735 (45 matching pairs of pancreatic tumor and adjacent non-tumor tissues). NCBI-GEO is a public repository containing an initial database of 1,325 biologically defined gene profiles and was developed to help in the analysis and interpretation of the long lists of genes produced from microarray-based experiments. The details of the series data are listed in Table 1.

**Table 1:** Detailed information of the five GEO data sets in the study.

**Series**	**Organism**	**Study type**	**Platform**	**Country**	**Samples**
GSE101448	*Homo sapiens*	Expression profiling by array	GPL10558 Illumina HumanHT-12 V4.0 expression beadchip	Germany	18 with pancreatic tumor and 13 non-tumor pancreatic tissue samples
GSE62165	*Homo sapiens*	Expression profiling by array	GPL13667 [HG-U219] Affymetrix Human Genome U219 Array	Belgium	118 surgically resected PDAC and 13 control samples
GSE62452	*Homo sapiens*	Expression profiling by array	GPL6244 [HuGene-1_0-st] Affymetrix Human Gene 1.0 ST Array [transcript (gene) version]	USA	69 pancreatic tumors and 61 adjacent non-tumor tissue from patients with pancreatic ductal adenocarcinoma
GSE41372	*Homo sapiens*	Expression profiling by array	GPL6244 [HuGene-1_0-st] Affymetrix Human Gene 1.0 ST Array [transcript (gene) version]	Italy	15 paired pancreatic tumor and adjacent non-tumor tissues
GSE28735	*Homo sapiens*	Expression profiling by array	GPL6244 [HuGene-1_0-st] Affymetrix Human Gene 1.0 ST Array [transcript (gene) version]	USA	45 matching pairs of pancreatic tumor and adjacent non-tumor tissues from 45 patients with pancreatic ductal adenocarcinoma

### Initial Identification of DEGs

As microarray batch effects appear to be the major contributors to the differential expression, SangerBox online tool (http://sangerbox.com/) was used to eliminate them before taken into account (15). Later, data were imported into the BRB-Array Tools to identify the DEGs between pancreatic tumor and normal pancreatic tissues. The Affymetrix platform (Santa Clara, California, USA) was applied for data set and gene annotation. The probes were filtered when some gene expression data were missing. The Bioinformatics and Evolutionary Genomics web tool (http://bioinformatics.psb.ugent.be/webtools/Venn/) was depicted graphically in the form of Venn diagram to detect the common DEGs among the five data sets.

### Establishment of FI Network

The Reactome pathway database (version 7.2.0; http://www.reactome.org) was applied to analyze the pathway enrichment for the sets of genes, investigate the functional relationships of the genes, and visualize the outcomes with diagrams that can be manually generated (16). Over 60% of human proteins were covered to help construct the Reactome FI network. The pairwise interaction information was uploaded into Cytoscape software (http://www.cytoscape.org) to establish the FI network (17). A false discovery rate (FDR) <0.05 was accepted as the cutoff criterion.

### Construction of the FI Network-Based Functional Modules

To construct the FI network-based module analysis, the Microarray Data Analysis bioinformatics tool installed in the ReactomeFIViz was applied (18). After uploading the text file containing gene expression data, correlations between genes in the FI-network were analyzed. The Markov Cluster algorithm (MCL) was used to achieve the functional modules with a selected module size. In order to supervise the size of the modules, we inputted 5.0 for the inflation coefficient (19). MCL modules in size of three or more were applied, and the average Pearson's correlation coefficient was ≥0.25. Six different colors were used to label the nodes in six different network modules. Module size ≥7 and fluorodeoxyglucose (FDG) <0.05 were used as cutoffs for the KEGG pathway and GO term enrichment analysis of the modules in a separate manner.

### Preliminary Validation of the Selected Gene

The Human Protein Atlas (http://www.proteinatlas.org) presented an expression map of human protein across 20 cancer types and 44 normal human tissues. It measured the RNA level as well as used antibody profiling to precisely localize the corresponding proteins (20). With the Human Protein Atlas (http://www.proteinatlas.org), we detected the IFITM1 protein expression in both pancreatic cancer and normal pancreatic tissues. Oncomine microarray database (http://www.oncomine.org) is a cancer microarray database as well as an online data-mining platform which contains 65 gene expression data sets comprising nearly 48 million gene expression measurements from over 4,700 microarray experiments (21). With the Oncomine online tool (http://www.oncomine.org), we explore the expression of IFITM1 in pancreatic cancer and normal pancreatic tissues across eight different data sets.

### Validation of IFITM1 With the Cancer Genome Atlas

Gene Expression Profiling Interactive Analysis (http://gepia.cancer-pku.cn/) is an online server, containing more than 8,000 normal and 9,000 tumor samples, providing interactive and customizable functions based on the Cancer Genome Atlas **(**TCGA) and Genotype-Tissue Expression (GTEx) database (22). Boxplot with jitter was applied to compare the expression level of IFITM1 mRNA in pancreatic cancer and paired normal pancreatic tissues. To validate the expression of IFITM1 correlating with survivals and pathological stages of patients with pancreatic cancer, the database was searched to obtain Kaplan–Meier survival curves and violin plots. The requested mRNA expressions above or below the median classified the patients into both high and low expression groups. The log-rank *p-*value and hazard ratio (HR) with 95% CIs were calculated and displayed on the website. A value of *p* < 0.05 was considered statistically significant.

### Immunohistochemical Staining

To analyze the IFITM1 expression by immunohistochemical (IHC) staining experiment, 90 patients with pancreatic cancer were included in the TMA analysis. Sample slides were dried for adherence and deparaffinized in xylene for decreasing ethanol concentrations. The tissue sections were marked and incubated with rabbit anti-IFITM1 diluted 1:1,500 (ab224063, Abcam, Cambridge, UK) at 4°C overnight. The percentage of the IHC staining and the staining intensity were assessed and recorded by two pathologists from the department of pathology, who were both blinded to the features of patients. The H-scores ranged from 0 to 100, where 100 represented 100% of regions with strong intensity. The H-scores were dichotomized by the cutoff and the median value for the statistical analysis.

### Cell Lines Culture and Short Hairpin RNA Lentiviral Transduction

Three human pancreatic cancer cell lines, PanC-1, BxPc-3, and SW1990, and one normal pancreatic cell line, HPDE6-C7, were used in the study. The panC-1 and BxPc-3 cells were purchased from the Cell Bank of Shanghai Institute of Biochemistry and Cell Biology (Shanghai, China), and the others were purchased from the American Type Culture Collection (ATCC, Manassas, VA, USA). The panC-1 and HPDE6-C7 cells were cultured in Dulbecco's modified Eagle's medium (DMEM) (Gibco, Brooklyn, NY, USA), and the BxPc-3 and SW1990 cells were cultured in Roswell Park Memorial Institute Medium (RPMI) 1640 (Gibco, Brooklyn, NY, USA) with both cultures supplemented with 10% fetal bovine serum (FBS; Gibco, Brooklyn, NY, USA) and 100 U penicillin/streptomycin (Gibco, Brooklyn, NY, USA) in a humid atmosphere containing 5% CO_2_ at 37°C. Short-tandem repeat (STR) profiles were authenticated to ensure the quality of the cell lines. All of the cells were passaged by standard cell culture techniques.

Two lentivirus-derived short hairpin RNAs (shRNAs) against IFITM1 were designed by Biolink Biotechnology Co. Ltd (Beijing, China) to silence the IFITM1 mRNA expression (the targeted sequences as follows: IFITM1 shRNA-1, 5′- CCTCATGACCATTGGATTCAT-3′; IFITM1 shRNA-2, 5′- CCTGTTCAACACCCTCTTCTT-3′. The procedure of transducing pancreatic cancer lines with shRNA lentiviral transduction was described in detail previously (23).

### Cell Viability Assay

Cells in logarithmic growth phase were seeded in 96-well Falcon Petri dishes (3,000 cells/well) the day before the assays were performed. The cell viability was assessed using a Cell Counting Kit (Dojindo, Kumamoto, Japan) based on sensitive colorimetric assays with the absorbance measured at 450 nm (OD 450 nm), according to the instructions of the manufacturer. The results were confirmed with three independent experiments.

### Quantitative Real-Time PCR Analysis

To extract total RNA, cells were lysed with the TRIzol Reagent (Invitrogen Corporation, Carlsbad, CA, USA) and reversed into complementary DNA (cDNA) with the QuantiTect Reverse Transcription Kit (TaKaRa, Dalian, China). The sequences of the designed primers used for the real-time PCR (qRT-PCR) experiments were as follows: IFITM1 forward, 5′-AGCCAGAAGATGCACAAGGA-3′ and reverse, 5′-GATCACGGTGGACCTTGGAA-3′; GAPDH forward, 5′- GAAGGTCGGTGTGAACGGATTTG-3′ and reverse, 5′- CATGTAGACCATGTAGTTGAGGTCA-3′ (Sangon, Shanghai, China).

### Western Blot Analysis

Radioimmunoprecipitation assay (RIPA; Invitrogen Corporation, Carlsbad, CA, USA) was added to protease, and phosphatase inhibitors were used to gain total protein samples. The proteins were separated by the sodium dodecyl sulfate-polyacrylamide gel electrophoresis (SDS-PAGE) gel (Bio-Rad, Hercules, CA, USA) and transferred to the polyvinylidene fluoride (PVDF) membrane (Sigma Chemical Corporation, St. Louis, MO, USA). The membranes were blocked for 1 h and then incubated overnight with primary antibodies against IFITM1 and β-actin (Abcam, Cambridge, UK), followed by the horseradish peroxidase (HRP)-linked secondary antibodies. SuperSignal West Femto Maximum Sensitivity Substrate (Thermo Fisher Scientific, Waltham, MA, USA) was used to detect the probed proteins.

### Wound Healing Assay

The cells were seeded in 48-well Falcon (Thermo Fisher Scientific Inc. Waltham, Massachusetts, USA) Petri dishes. Once 80–90% confluence was reached, a 200-μl sterile plastic pipette tip was used to make a single wound by gently scratching in every well. Cell debris was removed using phosphate-buffered saline and refreshed with serum-free medium immediately. Cells that extended the borders of the wound were photographed and quantified in three randomly selected regions per well.

### Colony Formation Assay

The cells in the logarithmic growth phase were seeded in 6-well Falcon Petri dishes (500 cells/well) and incubated for 7–14 days. The medium was refreshed every 3 days. The colonies were fixed with 70% ethanol and stained with crystal violet (0.5% w/v). The cell colonies containing at least 50 cells were photographed and quantified. The results were presented as mean ± SD for three independent experiments.

### Statistical Analysis

Statistical analyses were performed using SPSS 23.0 (SPSS Inc., Chicago, IL, USA) and Prism 8 (GraphPad Software, San Diego, CA, USA). Correlations between protein expression and clinicopathological features in patients with pancreatic cancer were analyzed by the χ^2^-test. Overall survival (OS) was evaluated using the Kaplan–Meier method and the log-rank test. A Cox proportional hazards regression model was used to assess the univariate and multivariate analyses. The Enter method was used to select the independent variables in the multivariate analysis.

## Results

### Screening of DEGs

**Figure 2:**
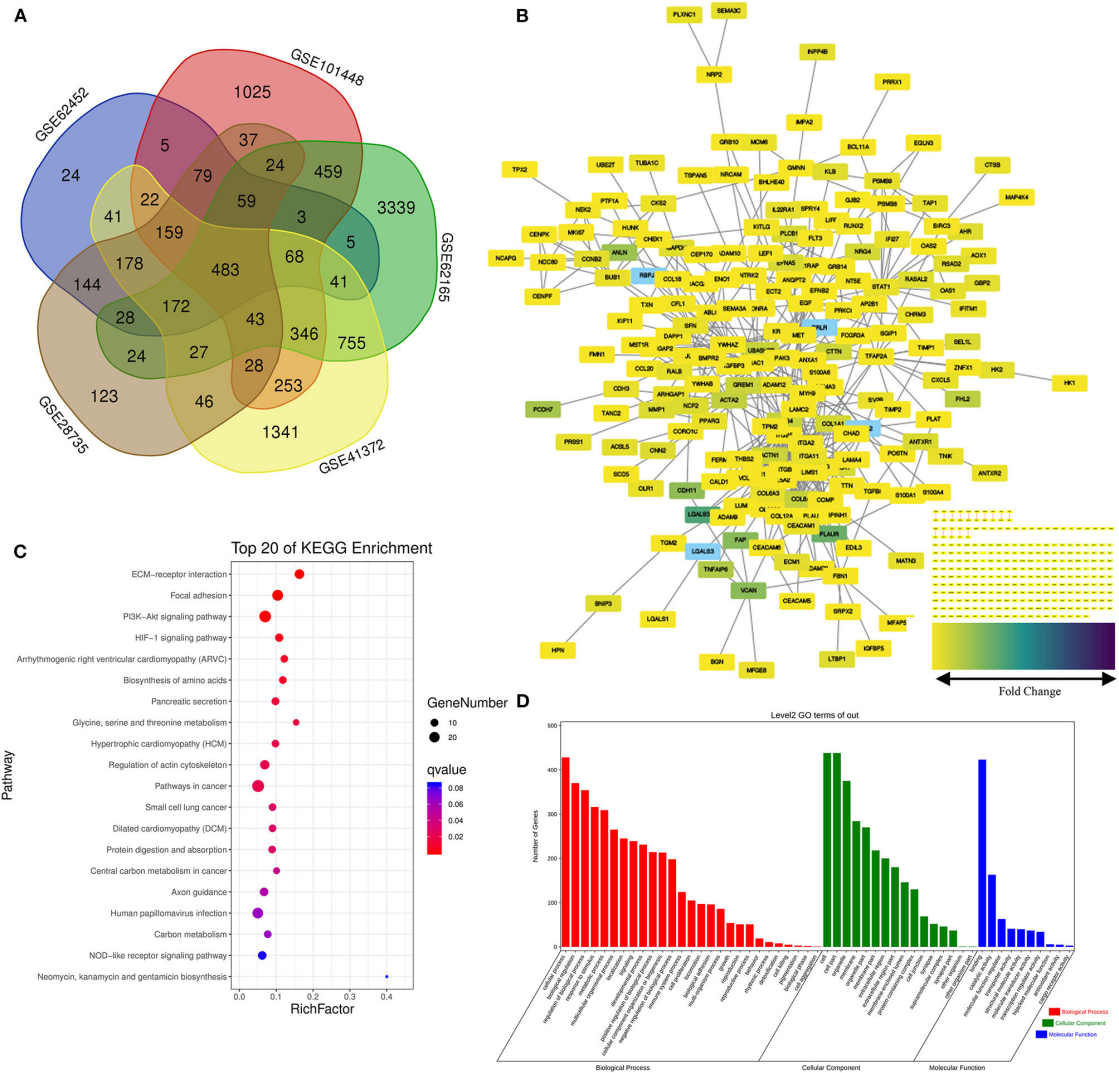
Screening of the differentially expressed genes (DEGs) in the pancreatic cancer establishment of the functional interaction (FI) network. **(A)** A flower-shaped Venn diagram showing 483 DEGs between pancreatic cancer and normal pancreatic tissues among five studies. **(B)** The FI network was established using pancreatic cancer-related 483 DEGs. Edges were based upon the FI annotation. Node colors defined fold changes in pancreatic cancer-related DEGs, ranging from yellow for low expression to purple for high expression, compared to non-cancerous samples. **(C,D)** Gene functional enrichment of the 483 DEGs. Kyoto Encyclopedia of Genes and Genomes (KEGG) showed the top 20 signaling pathways, and Gene Ontology (GO) analysis showed the biological processes and molecular functions involved in differential genes.

Five data sets from GEO containing gene expression profiles of pancreatic cancer as well as normal pancreas samples were downloaded for microarray analysis. The random variance mode method along with the paired *t*-test was applied for the differential expression calculation. Considering the criteria of |log2 (Fold change) |>1 adjusted *p* < 0.05, 483 DEGs were obtained (Figure 2A). Of those, 231 DEGs were identified upregulated and 252 downregulated (Supplementary Table 1).

### Establishment of the Pancreatic Cancer-Related FI Network

Functional interaction data derived from the Human Protein Reference Database (http://www.hprd.org/) (24), BioGrid (https://thebiogrid.org/) (25), I2D (http://ophid.utoronto.ca/) (26), IntACT (https://www.ebi.ac.uk/intact/) (27), MINT (https://www.ebi.ac.uk/intact/) (28), the Database of Interacting Proteins (http://dip.doe-mbi.ucla.edu) (29) as well as multiple high-throughput assays (30). We established the FI network by mapping the pancreatic cancer-related DEGs to the FIs data. Of 245 isolated nodes, 227 in eight clusters were identified with an effective mean degree of 2.20 (Figure 2B). The connectivity of a node represents the number of its neighbors, and the neighborhood connectivity represents the average connectivity of all neighbors of the selected node. Under the criteria of neighborhood connectivity ≥20 and the shortest path length ≥3.5 for non-single modules, eight genes (*EDIL3, RSAD2, AHR, GBP2, IFITM1, AOX1, KIF11*, and *CEP170*) among all the nodes in the FI network were filtered out (Supplementary Table 2).

### Functions Analysis of the FI Network

To gain insights into the role of the putative targets involved in various BPs and molecular functions (MFs), the preliminary GO function and KEGG pathway analysis were performed using the OmicShare online database. As expected, among the top 20 KEGG pathways, extracellular matrix (ECM)–receptor interaction, pancreatic secretion, and pathways in cancer had significant relations to tumorigenesis and progression of pancreatic cancer (Figure 2C; for details see Supplementary Table 3). Among the most highly enriched functions in the BP category, cell process, biological regulation, and regulation of BP were found associated with the tumorigenesis and progression of pancreatic cancer. Meanwhile, cell, cell part, organelle, and membrane were top enriched items in the cellular component (CC) category. In the MF category, the DEGs were mainly enriched in binding activities (Figure 2D; for details see Supplementary Tables 4–6).

### Identification of FI Network-Based Functional Modules

**Figure 3:**
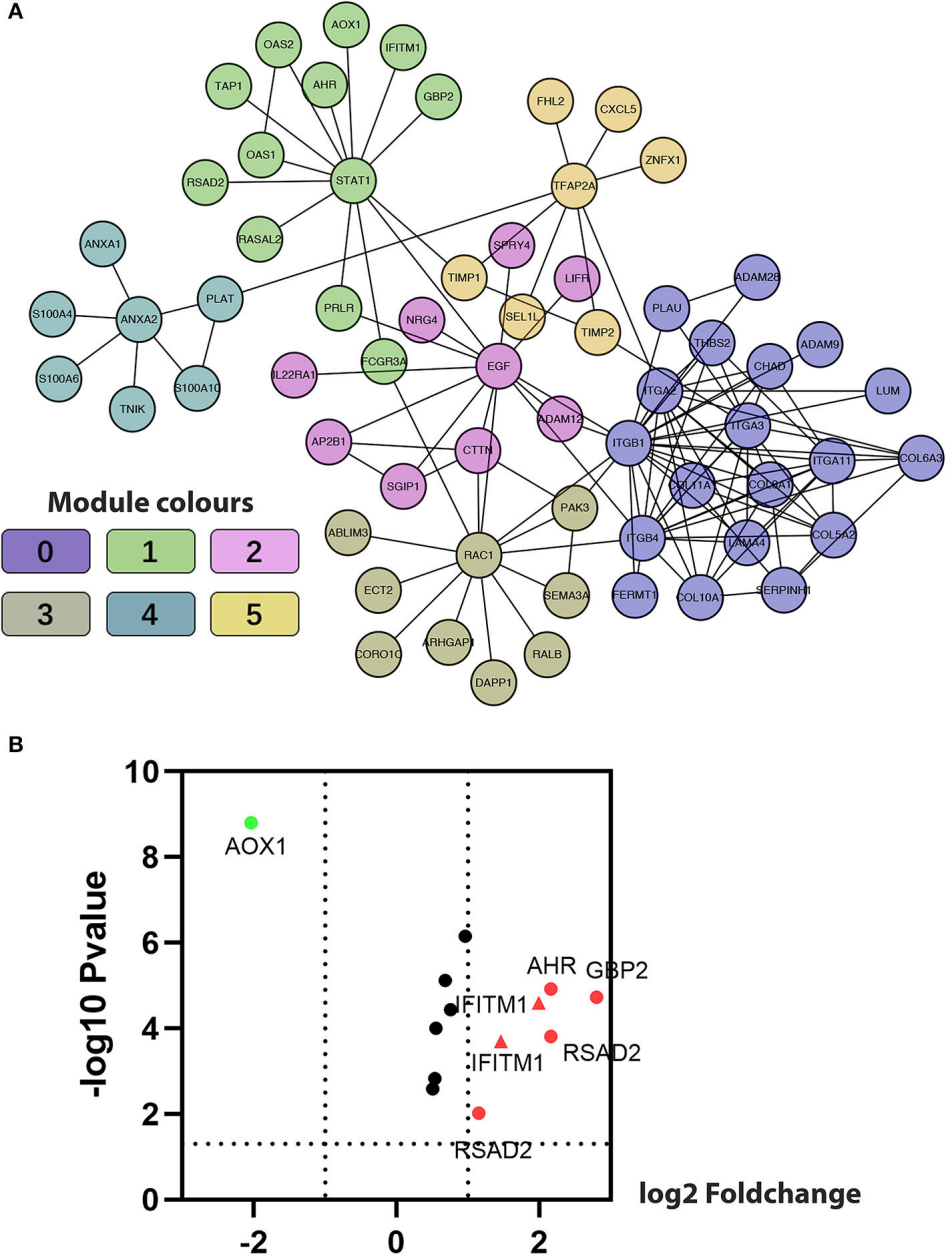
Construction of functional interaction (FI) network-based module genes. **(A)** A functional network constructed by six FI-based modules comprises 23 genes in different colors. **(B)** A volcano map shows differentiating pancreatic cancer samples from normal samples, with red representing significantly upregulated genes, green representing significantly downregulated genes, and the red triangle representing IFITM1.

The Markov Cluster algorithm (MCL) was applied for the network-based module-clustering algorithm, and the absolute value of the Pearson's correlation coefficient of the interacting genes was used to weigh each correlation edge. A total of 6 modules involving 63 genes were obtained, ranging from 7 to 19 genes per module (Table 2, Figure 3A). Later, GO function and pathway analyses were performed on the six network-based functional modules. Through enrichment analysis (Supplementary Table 7), we identified that Module 0 containing 19 genes was in relation to the classical cell–cell adhesion process. Module 1 was of the highest average correlation and was related to the classical cancer signaling pathway in the KEGG and IFN-gamma pathway in NCI-PID. Module 2 was found related to cell motility and proliferation in KEGG and Module 3 to cell migration and cytoskeleton. Fibro-relevant families in Reactome were enriched in Module 4. Meanwhile, the enrichment of GO term was also analyzed. The calculation results showed that the GO functions of six modules complied with the pathway annotations (Supplementary Tables 8–10).

**Table 2:** Genes analyzed in six modules in the FI network.

**Module number**	**Number of genes**	**Average correlation**	**Gene set**
0	19	0.9927	ADAM28, ADAM9, CHAD, COL10A1, COL11A1, COL5A2, COL6A3, COL8A1, FERMT1, ITGA11, ITGA2, ITGA3, ITGB1, ITGB4, LAMA4, LUM, PLAU, SERPINH1, THBS2
1	12	1.0000	AHR, AOX1, FCGR3A, GBP2, IFITM1, OAS1, OAS2, PRLR, RASAL2, RSAD2, STAT1, TAP1
2	9	0.9615	ADAM12, AP2B1, CTTN, EGF, IL22RA1, LIFR, NRG4, SGIP1, SPRY4
3	9	0.8365	ABLIM3, ARHGAP1, CORO1C, DAPP1, ECT2, PAK3, RAC1, RALB, SEMA3A
4	7	0.9739	ANXA1, ANXA2, PLAT, S100A10, S100A4, S100A6, TNIK
5	7	0.7790	CXCL5, FHL2, SEL1L, TFAP2A, TIMP1, TIMP2, ZNFX1

Five genes (*IFITM1, AHR, AOX1, GBP2*, and *RSAD2*) common to both Module 1, with an average correlation equal to 1.00, and the set of previously selected eight genes were selected for the volcano plot analysis of the five GEO data sets (Figure 3B, IFITM1 marker in the red triangle). Through comprehensive literature searching, studies relative to AHR and pancreatic cancer have been taken, but not enough background data was found about AOX1, GBP2, and RSAD2 (31). Combining the results from the functional analysis of the FI network and literature mining, we were interested to see if IFITM1 could be a potential biomarker in pancreatic cancer.

### Aberrant Expression of IFITM1 in Pancreatic Cancer

**Figure 4:**
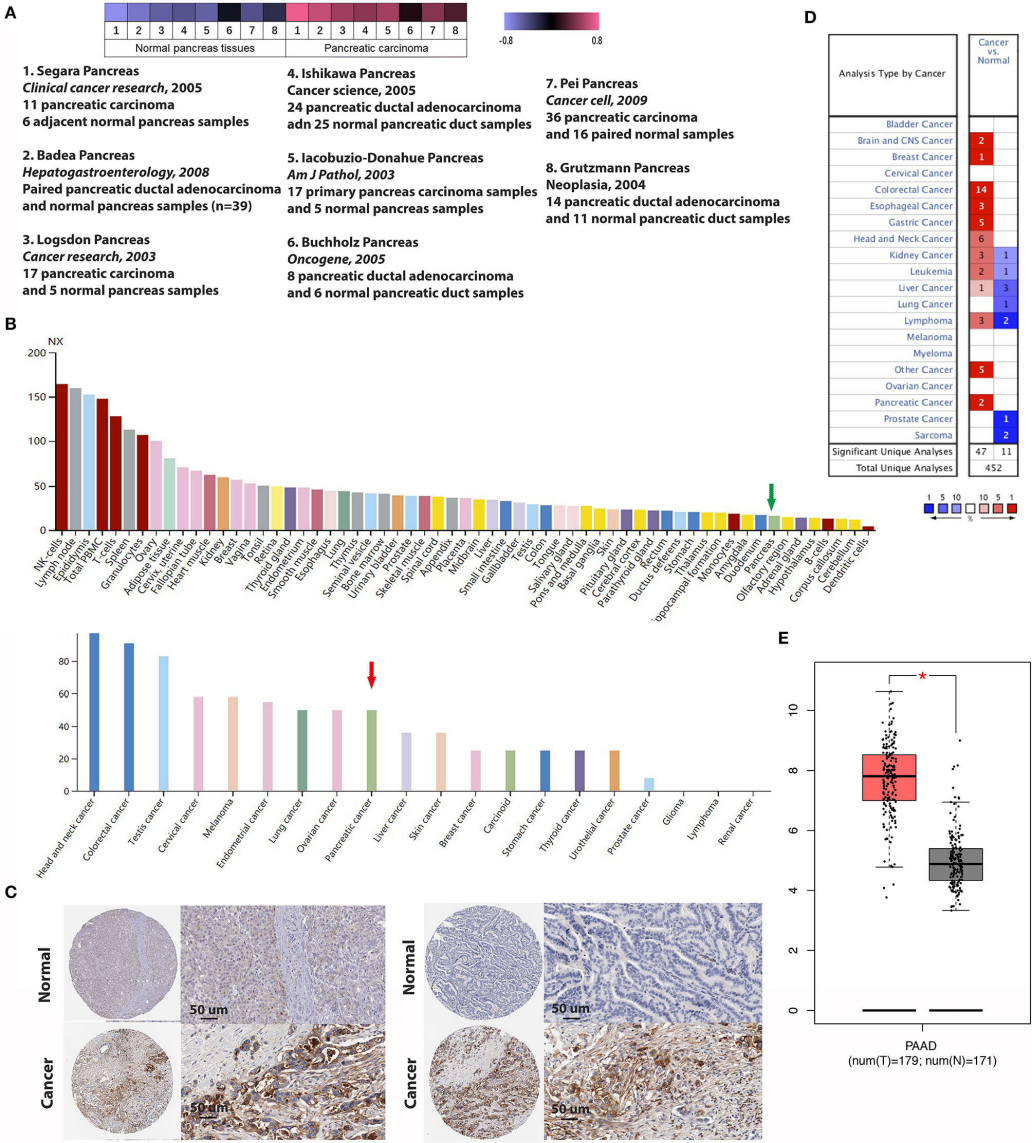
The expression of IFN-induced transmembrane protein 1 (IFITM1) between pancreatic cancer and normal pancreas samples. **(A)** Heat map of IFITM1 associated with pancreatic cancer among eight studies. **(B,C)** An overview of IFITM1 levels in pancreatic tumors and normal pancreatic tissues and immunohistochemical analysis of IFITM1 expression in pancreatic cancer and normal pancreatic tissues. Data were derived from the Human Protein Atlas database. **(B)** The green and the red arrows represent normal pancreatic tissue and pancreatic cancer, respectively. **(C)** Top: Protein levels of IFITM1 in normal tissue (staining: negative; intensity: negative; quantity: none); bottom: Protein levels of IFITM1 in pancreatic cancer tissue (staining: high; intensity: strong; quantity: >75%). **(D)** mRNA levels of IFITM1 in 20 types of cancers vs. normal tissues. The figure represented the number of data sets involving statistically significant upregulated (dark) as well as downregulated (light) expression of IFITM1. **(E)** The box plot shows the expression profile of the IFITM1 based on GEPIA. The red node represents pancreatic cancer, and the gray node represents normal samples. *p < 0.05.

First, we used the Oncomine database to explore the transcriptional level of IFITM1 in pancreatic cancer and normal pancreatic tissues (Figure 4D). Based on the data from the Oncomine database, the transcriptional level of IFITM1 was significantly elevated in pancreatic cancer tissue vs. normal pancreatic tissue. Same results can also be seen in colorectal, esophageal, gastric, liver, and many other cancer types. Eight studies reported from 2003 to 2009 were filtered out with the screening condition of “IFITM1; Cancer vs. Normal Analysis; Cancer Type: Pancreatic Cancer” and assessed with a heat map analysis (Figure 4A). Segara et al. (32) found that the level of IFITM1 in pancreatic cancer was significantly increased compared to adjacent normal pancreas samples, with a fold change of 5.521 and a *p-*value of 6.78E-7. The same results were also reported by Badea et al. (33) (fold change of 3.065 and *p*-value of 8.21E-9), Logsdon et al. (34) (fold change of 4.104 and *p*-value of 1.14E-4), Ishikawa et al. (35) (fold change of 2.350 and *p*-value 0.008), and Iacobuzio-Donahue et al. (36) (fold change of 2.855 and *p*-value of 0.005), respectively. While Buchholz et al. (37) and Grutzmann et al. (38) found no significant upregulation in pancreatic cancer with *p-*values of 0.412 and 0.231, respectively. Recently, Pei et al. (39) studied 36 samples of pancreatic carcinoma and 16 paired normal samples, and statistical significance was reported with a fold change of 2.304 and a *p-*value of 0.002. Meanwhile, we found the protein expression of IFITM1 with the help of the Human Protein Atlas, and the positively strong level was also found in pancreatic cancer specimens compared with normal tissues (Figures 4B,C). Furthermore, the GEPIA online tool (https://www.gepia.cancer-pku.cn) was also used to validate the differential mRNA expression analysis of IFITM1 in pancreatic cancer and normal tissues (Figure 4E).

**Figure 5:**
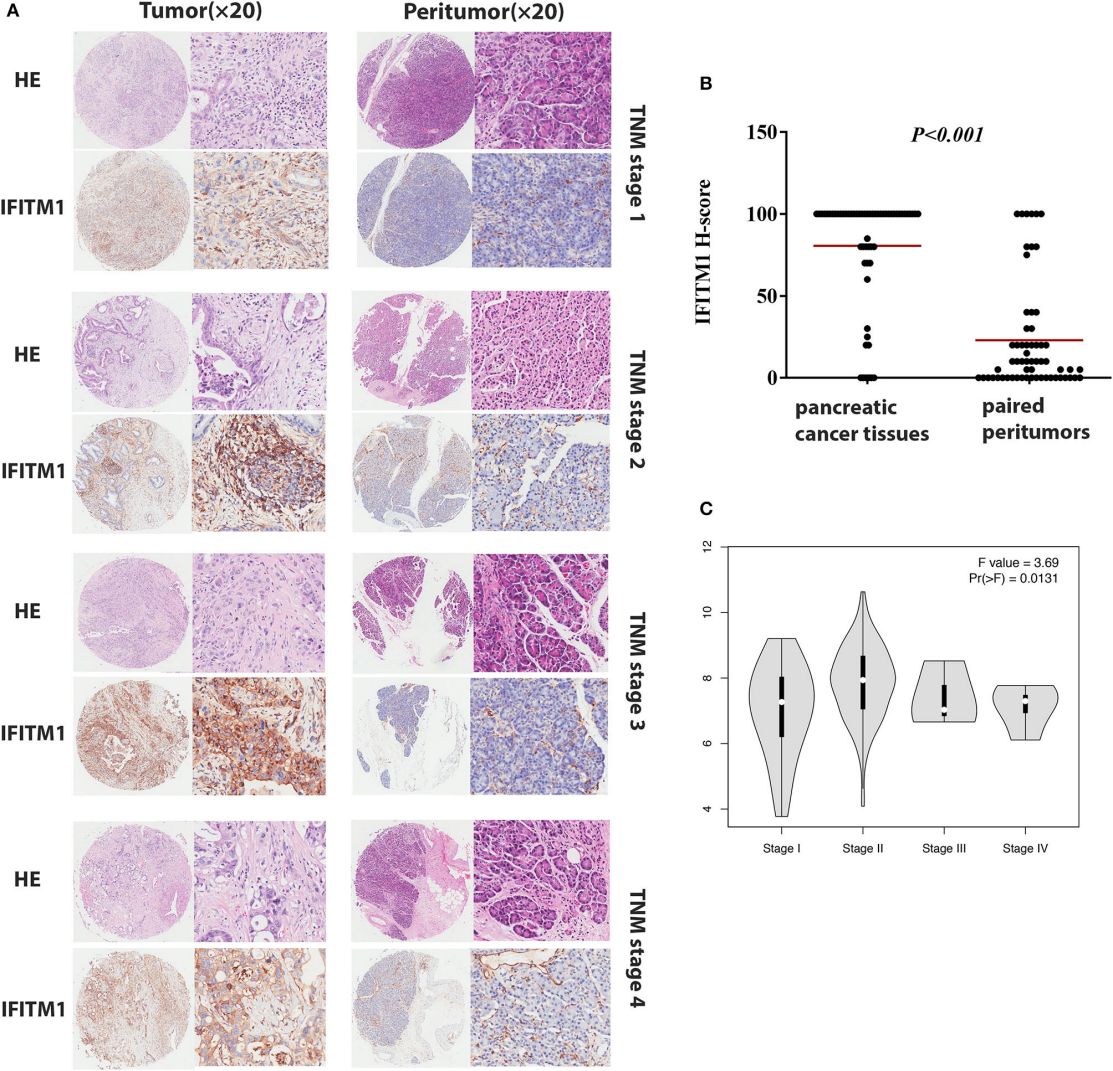
The IFN-induced transmembrane protein 1 (IFITM1) expressions in pancreatic cancer samples and paired peritumor tissues. **(A)** IFITM1 in representative cases of pancreatic cancer tissues in TNM stage 1–4 were detected by IHC experiments, respectively. Original magnification × 20. **(B)** The differential protein expression of IFITM1 was shown in pancreatic cancer tissues and matched non-tumor tissues of 90 patients as indicated. The median extents of the expressions were indicated by the horizontal line in the scatterplot figure. **(C)** Correlations between different expressed IFITM1 and the pathological TNM stage of pancreatic cancer patients.

Immunohistochemical experiments were applied to validate IFITM1 expression in 90 patients with pancreatic cancer. Representative IHC-stained images showed that IFITM1 is mainly located in the cell membrane of the pancreatic cancer tissues. A significantly upregulated expression of IFITM1 was found in 76.7% of the patients (69/90), which was consistent with the results analyzed by the microarray data (*p* < 0.001, see Figures 5A,B).

### Correlation of IFITM1 Expression of Clinical Features in Patients With Pancreatic Cancer

The χ^2^-test was applied to assess the relationship between the expressions of IFITM1 and the clinical and pathologic features of patients with pancreatic cancer, including gender, age at diagnosis, tumor size, histologic differentiation, TNM stage, nodal status, and distant metastasis. Histologic differentiation (*p* = 0.032) and TNM stage (*p* = 0.044) were found to have close correlations with IFITM1 expression (Table 3). Higher expression of IFITM1 correlated with poor histologic differentiation and larger TNM stage. The significant correlation between IFITM1 and the TNM stage was also verified using the GEPIA (https://www.gepia.cancer-pku.cn; Figure 5C). Consistent with our investigation, IFITM1 might be an oncogene for pancreatic cancer that participates in tumorigenesis.

**Table 3:** Relationship between the expression of IFITM1 and clinical characteristics of 90 patients with pancreatic cancer.

**Parameter**	**Patients**		**IFITM1 expression**		
	**Number**	**Percentage**	**Low**	**High**	***p*-value**
Gender					0.808
Male	58	64.44	14	44	
Female	32	35.56	7	25	
Age at diagnosis (year)					0.944
≤ 65	54	60.00	13	41	
>65	36	40.00	9	27	
Tumor size (cm)					0.839
≤ 4.5	54	60.00	13	41	
>4.5	36	40.00	8	28	
Histologic differentiation					0.032
Well	9	10.00	4	5	
Moderate	48	53.55	14	34	
Poor	33	36.67	3	30	
TNM stage					0.044
1	3	3.33	2	1	
2	66	72.22	12	54	
3	18	21.11	7	11	
4	3	3.33	1	2	
Nodal status					0.970
negative	50	55.56	11	40	
1	33	36.67	8	26	
2	7	7.78	2	3	
Distant metastasis					0.259
negative	86	95.56	21	0	
positive	4	4.44	65	4	
TNM Stage					0.512
I	3	3.33	1	2	
II	37	41.11	6	31	
III	42	46.67	11	31	
IV	8	8.89	3	5	

### The Cancer Genome Atlas-Based Analysis Validates the Prognostic IFITM1 Signature

**Figure 6:**
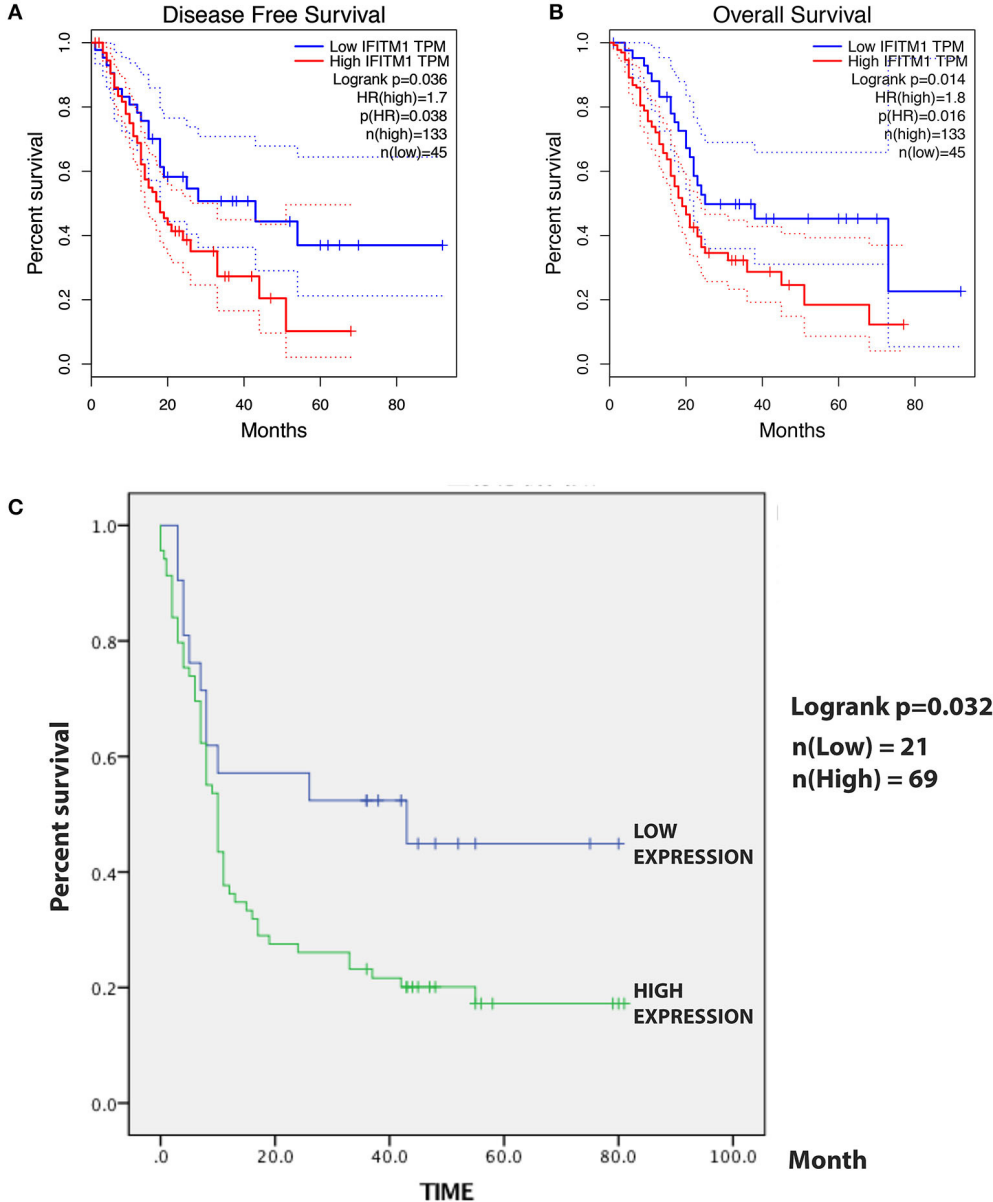
The prognostic value of different expressed IFITM1 in patients with pancreatic cancer. **(A)** Kaplan–Meier curve for the disease-free survival of patients with pancreatic cancer with low vs. high expression of IFITM1 [median progression-free survival (PFS) <20 months vs. more than 20 months, respectively; log-rank test, *p* = 0.036]. **(B)** Kaplan–Meier curve for the overall survival (OS) of patients with low vs. high expression of IFITM1 (log-rank test, *p* = 0.016). **(C)** Kaplan–Meier curve for the 90 patients with pancreatic cancer in our study.

A total of 184 patients with pancreatic cancer in the TCGA database, with 5 of them having no documented T or N stage, leaving 179 patients for the confirmation of the relationship between IFITM1 and human pancreatic cancer. The Kaplan–Meier analysis and the log-rank test were used for analysis. As shown in Figure 6A, patients with a low level of IFITM1 had better disease-free survival than those with high IFITM1 (log-rank *p* = 0.036) as well as had a better OS (Figure 6B, log-rank *p* = 0.014).

### Verification of IFITM1 With Prognostic Signature

For the survival analysis, the median IHC value was used as an objective cutoff to stratify 90 patients with pancreatic cancer with low and high expressions. We used the Kaplan–Meier analysis and the log-rank test to explore the correlation. The follow-up time was 81 months, and a high IFITM1 level was found significantly related to decreased OS (*p* = 0.032; Figure 6C). The median OS of the patients with pancreatic cancer with the low and high IFITM1 expression was 43.0 vs. 10.0 months, respectively.

As for the univariable Cox regression analysis, positive nodal status (*p* = 0.008), TNM stage (*p* = 0.012), histologic differentiation (*p* = 0.004), and IFITM1 expression (*p* = 0.040) were significantly associated with worse outcome (Table 4). In the multivariable analysis, IFITM1 expression (HR = 2.134; 95% CI 1.073, 4.245; *p* = 0.031) remained an independent prognostic marker for OS of patients with pancreatic cancer (Table 4).

**Table 4:** Univariable and multivariable Cox regression analyses for overall survival, accounting for IFITM1 expression and clinicopathological features in 90 patients with pancreatic cancer.

**Parameter**	**Univariable**		**Multivariable**	
	**Hazard ratio (95% CI)**	***p*-value**	**Hazard ratio (95% CI)**	***p-*value**
Gender (female/male)	1.164 (0.702, 1.931)	0.556		
Age (years; ≤ 65/>65)	1.200 (0.733, 1.963)	0.469		
TNM stage (1/2/3/4)	1.541 (0.939, 2.529)	0.087		
Nodal status (0/1/2)	1.670 (1.140, 2.447)	0.008	1.026 (0.535, 1.970)	0.938
Distant metastasis (-/+)	2.548 (0.921, 7.053)	0.072		
TNM Stage(I/II/III/IV)	1.563 (1.103, 2.215)	0.012	1.684 (0.886, 3.198)	0.111
Tumor size (≤4.5/>4.5 cm)	1.212 (0.739, 1.990)	0.446		
Histologic differentiation (well/moderately/poorly)	1.819 (1.214, 2.725)	0.004	1.616 (1.060, 2.462)	0.026
IFITM1 expression (≤median/>median)	1.971 (1.030, 3.771)	0.040	2.134 (1.073, 4.245)	0.031

### Immune Cell Infiltration of IFITM1 in Patients With Pancreatic Cancer

**Figure 7:**
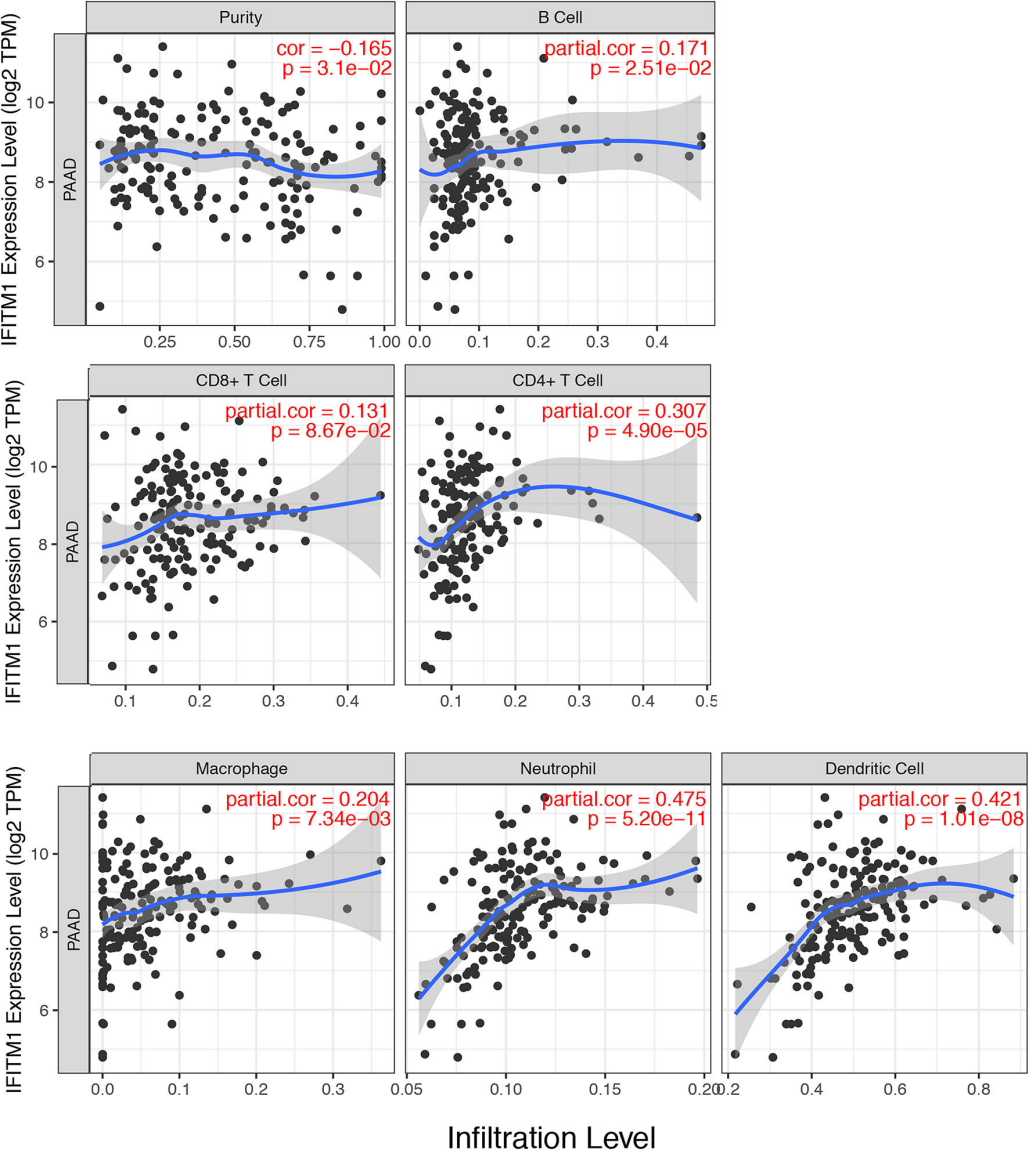
The correlations between different IFITM1 expression and the immune cell infiltration (TIMER) in pancreatic cancer.

IFN-induced transmembrane protein 1 was found involving in inflammatory processes and immune cells infiltration, which might further influence the clinical outcomes of patients with pancreatic cancer. We used the TIMER database (https://cistrome.shinyapps.io/timer/) to explore a comprehensive relation between IFITM1 levels and immune infiltrations. The positive correlations were assessed between IFITM1 and B cell (Cor = 0.171, *p* = 2.51E-2), CD4+ T cell (Cor = 0.307, *p* = 4.90E-5), macrophage (Cor = 0.204, *p* = 7.34E-3), neutrophil (Cor = 0.475, *p* = 5.20E-11), and dendritic cell (Cor = 0.421, *p* = 1.01E-8). IFITM1 level was in negative association with purity (Cor = −0.165, *p* = 3.1E-2). Only CD8+ T cell was found having no significant relation to IFITM1 expression (Figure 7).

### IFN-Induced Transmembrane Protein 1 Silencing Inhibits Tumorigenicity of Pancreatic Cancer

**Figure 8:**
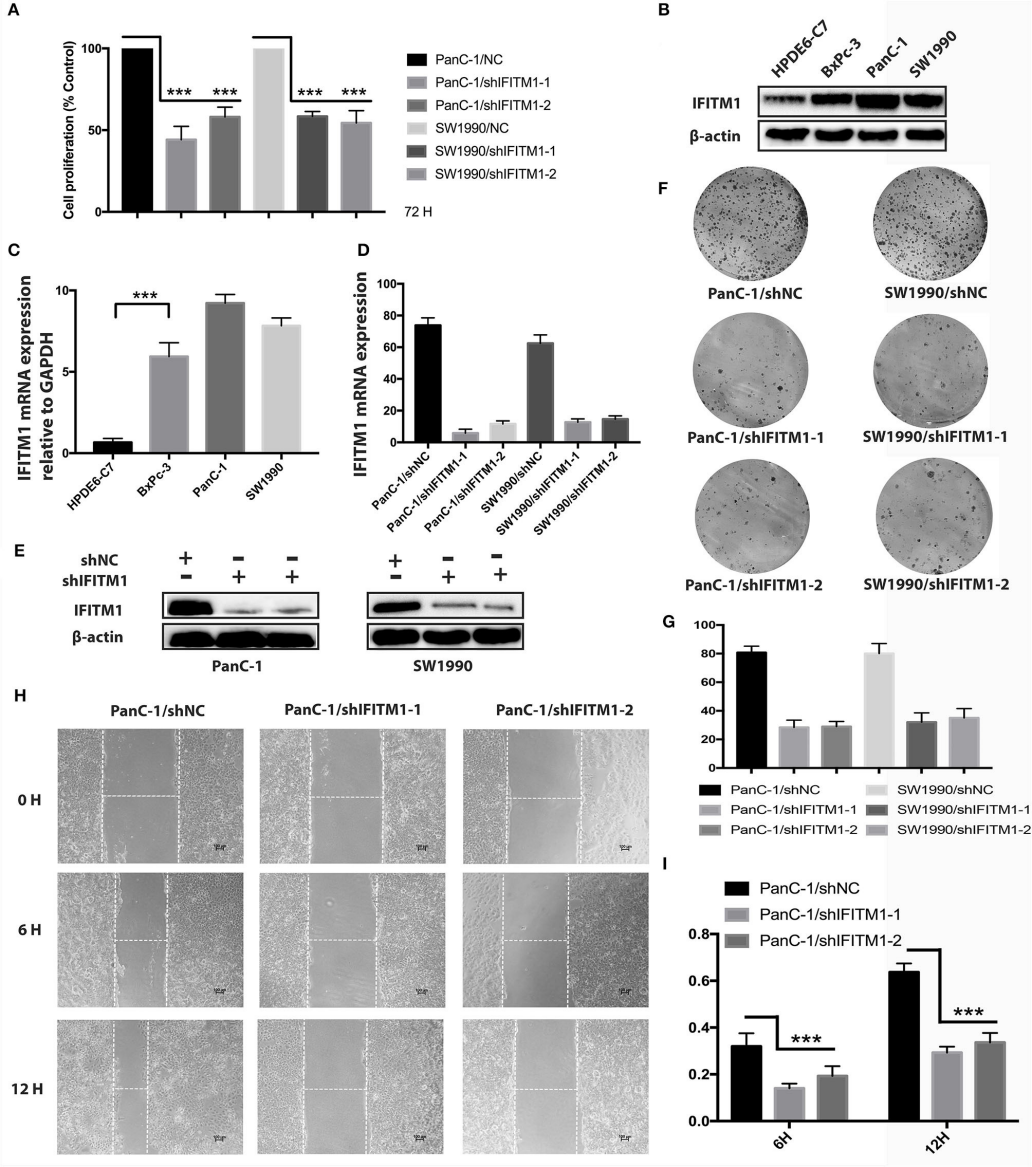
The depletion of IFITM1 decreased the tumorigenicity of pancreatic cancer cells. **(A)** Control (shNC) or IFITM1-depleted (shIFITM1-1 and shIFITM1-2) PanC-1 and SW1990 cell lines were incubated for 72 h to determine the cell proliferation rate by cell counting kit-8 assay. Data represent the mean ± SD of three independent experiments (***p < 0.001). **(B,C)** Detection of IFITM1 mRNA expression in three different pancreatic cancer cell lines and HPDE6-C7 by qRT-PCR and Western blot analysis. GAPDH and β-Actin were used as controls, respectively. The results are expressed as the mean ± SD of three independent experiments (***p < 0.001). **(D,E)** Pancreatic cancer cell lines were transfected with shRNAs (shIFITM1-1 and shIFITM1-2), and the expression level of IFITM1 was assessed by qRT-PCR and Western blot analysis using GAPDH and β-actin as controls, respectively. The results are expressed as mean ± SD of three independent experiments. **(F,G)** Colony-formation assay was performed to show decreased cancer stem cell-like properties of PanC-1 and SW1990 cells transfected with shIFITM1-1 or shIFITM1-2 compared with control *in vitro*. **(H,I)** The wound-healing assay was performed to assess the effect of IFITM1 silencing on the wound closure ability of PanC-1 cells. Representative images are shown (magnification ×100).

Three types of pancreatic cancer cell lines and normal human pancreatic duct epithelial cell line HPDE6-C7 were used to examine the expressions of IFITM1. As shown in Figures 8B,C, higher mRNA and protein levels of IFITM1 were found in BxPc-3, PanC-1, and SW1990 compared to HPDE6-C7. Two shRNA targeting IFITM1 were transfected into PanC-1 and SW1990 to detect the IFITM1 silencing effects, and the efficiency was examined both by qRT-PCR and Western blot analysis (Figures 8D,E). We performed cell counting kit-8 assay to assess the cancer cell proliferation. The results showed that the downregulation of IFITM1 significantly decreased the proliferation of PanC-1 and SW1990 cell lines (Figure 8A). Moreover, the wound-healing assay indicated that IFITM1 silencing inhibited cell migration capability, and the colony-formation assay showed that it greatly reduced the cancer stem cell-like properties of pancreatic cancer (Figures 8F–I).

## Discussion

With the highest incidence-to-mortality ratio of solid tumors, pancreatic cancer is an elusive disease seeking explorations to elicit the redundant mechanisms involving pathogenesis, progression, and metastasis. To improve the poor clinical expectations, we identified and verified a potential molecular biomarker through network-based analytic methods of multiple data sets and etiology-based malignancies characterizing (30).

Systematically, through five microarray gene expression data sets, the FI network-based approach was used to build the functional modules for pancreatic cancer. On the basis of the diverse function of each module, the degree value, and the connectivity of the nodes, IFITM1 was identified as a promising biomarker. Consistent with the outcomes of several bioinformatics online tools, IFITM1 was found highly expressed in pancreatic cancer compared to normal pancreatic tissues and was confirmed through IHC experiment later. Similarly, previous studies have shown high expressed IFITM1 in a variety of cancers. Through the human cancer profiling array containing 10 samples of lung carcinoma and the corresponding normal samples, IFITM1 was detected increasing significantly in lung carcinoma (40). The difference was also observed in two other studies that demonstrated IFITM1 overexpression through tissues and lung cancer cell lines of patients (8, 41). Immunohistochemistry on TMA of a consecutive cohort including 174 patients with gastroesophageal adenocarcinoma also detected a significant elevation of IFITM1 in primary tumors along with lymph node metastases, compared to the adjacent normal epithelium and intestinal metaplasia (42). Gastric cancer samples derived from 27 patients and 6 gastric cancer cell lines, and tissues of 4 patients and 4 tumor cell lines were assessed by Lee et al. (7) and Yang et al. (43) separately and showed the same findings. Moreover, aberrant expressions of IFITM1 detected through cancer array profiling, immunohistochemistry, RT-PCR, immunoblotting, and immunofluorescence analysis were verified in colorectal cancer, hepatocellular carcinoma, head and neck squamous cell carcinoma (HNSCC), gallbladder, and ovarian cancer (6, 8, 23, 44–46).

Notably, the histologic differentiation (*p* = 0.032) and TNM stage (*p* = 0.044) of the patients with pancreatic cancer were significantly correlated with IFITM1 expression in the clinical and pathological analyses. In order to explore the underlining mechanisms, we found the role of IFITM1 has been shown in the tumorigenesis involving tumor cells unlimited proliferation, invasion, angiogenesis, metastasis, and treatment resistance. Overexpression of IFITM1 was for the first time found to enhance cancer migration and invasion in 2005, with the cell model of gastric cancer (43). The phenomenon was then discovered in HNSCC, glioma, and gastric cells *in vitro* experiments, and meanwhile the downregulation of IFITM1 significantly decreased the activity of matrix metalloproteinase 9 (MM9) (44, 47). Two studies revealed that the depletion of IFITM1 significantly inhibited aromatase inhibitor (AI)-resistant breast cancer growth, and the invasion and observation reversed with the ectopic level of IFITM1 (48, 49). Recently, Yang et al. (43) demonstrated that IFITM1 was essentially required for the progression of non-small cell lung cancer (NSCLC), both *in vitro* and *in vivo* and the silenced expression of IFITM1 significantly reduced the capacity of sphere-formation, invasion, and migration of NSCLC.

To further validate the prognostic signature of IFITM1 in patients with pancreatic cancer, both univariable and multivariable Cox regression analyses were assessed, and the data suggested IFITM1 as an independent prognostic marker. Similar prognostic values were also seen in other types of tumors (42, 45, 50). Chemotherapy, radiation therapy, and endocrine therapy are three critical and effective strategies in cancer treatment. Accumulating evidence has indicated that IFITM1 was involved in radio- and chemo-resistance as well as endocrine therapy resistance. In our previous study, we explored the radiosensitive possibility of adipose-derived mesenchymal stem cells and discovered IFITM1 could be an important target involved in radioresistance (23). Mechanically, the upregulation level of IFITM1 was correlated with high expression of STAT3 and MMP family and also with the downregulation of P53 and caspase family. The association between IFITM1 and signal transducer STAT at the molecular level was also detected and evaluated by knockdown of IFITM1 in oral tumors (51, 52). While the interaction between IFITM1 and P53 signaling cascades, the apoptotic effects of other IFITM proteins were mainly P53-independent (53). Yet, it is still unclear whether IFITM1 is of prime importance in the responsiveness of patients with pancreatic cancer to clinical therapies.

In addition to the multiple cell functions involving anti-proliferation and adhesion, the IFITM1 is predominantly important in immune responses (54). As we discovered above, immune cell infiltration was in significant correlation with IFITM1 in pancreatic cancer. Escape of natural killer cell recognition was reported from gastric cancer cells with an increased level of IFITM1 (43). This indicated that tumors expressing high IFITM1 were insensitive to the antiproliferative therapies, which might be attributed to escaping immune surveillance.

Although we discovered the potential role of IFITM1 in pancreatic cancer, limitations to this study were acknowledged. A tumor xenograft model is needed to verify the outcomes *in vivo* experiments, and a larger cohort of patients with pancreatic cancer are expected to validate the data.

Taken together, our study constructed an effective FI network-based module analysis to discover the differentially expressed genes between pancreatic cancer and normal pancreatic tissues and suggested that the upregulation of IFITM1 correlated to a poor clinical result of pancreatic cancer.

## Data Availability Statement

The original contributions presented in the study are included in the article/Supplementary Material, further inquiries can be directed to the corresponding author/s.

## Ethics Statement

The studies involving human participants were reviewed and approved by the Ethical Committees of the National Engineering Center for Biochips at Alenabio and Shanghai Outdo Biotech Company and Taizhou People's Hospitals. The patients/participants provided their written informed consent to participate in this study. The animal study was reviewed and approved by the Ethical Committees of the National Engineering Center for Biochips at Alenabio and Shanghai Outdo Biotech Company and Taizhou People's Hospitals. Written informed consent was obtained from the individual(s) for the publication of any potentially identifiable images or data included in this article.

## Author Contributions

SY and CM were responsible for the research design and participated in the paper. LW and XZ drafted the manuscript and participated in data collection and analysis. LW, XZ, MT, and DY performed the laboratory experiments. All authors were responsible for data analysis and figure format.

## Conflict of Interest

The authors declare that the research was conducted in the absence of any commercial or financial relationships that could be construed as a potential conflict of interest.

## Abbreviations

IFN: interferon
IFITM1: IFN-induced transmembrane protein 1
ISGs: IFN-stimulated genes
GEO: Gene Expression Omnibus
DEGs: differentially expressed genes
FI: functional interaction
GO: Gene Ontology
KEGG: Kyoto Encyclopedia of Genes and Genomes
FDR: false discovery rate
TCGA: the Cancer Genome Atlas
IHC: immunohistochemical
MCL: Markov cluster algorithm
NSCLC: non-small cell lung cancer
TMA: tissue microarray
BP: biological process
MF: molecular function
HNSCC: head and neck squamous cell carcinoma
OS: overall survival.
